# Synergistic Role of Crosslinker and Silane-Based Additive in Designing Structurally Robust Bio-Based Polyurethane Coatings

**DOI:** 10.3390/polym18121490

**Published:** 2026-06-13

**Authors:** Mayankkumar L. Chaudhary, Kinal Chaudhari, Rutu Patel, Ram K. Gupta

**Affiliations:** 1 National Institute for Materials Advancement, Pittsburg State University, 1204 Research Road, Pittsburg, KS 66762, USA; kinalkumarrohitkumar.chaudhari@gus.pittstate.edu (K.C.); rutupatel114538@gmail.com (R.P.); 2 Department of Physics, Pittsburg State University, 1701 S Broadway St, Pittsburg, KS 66762, USA; 3 Department of Chemistry, Pittsburg State University, 1701 S Broadway St, Pittsburg, KS 66762, USA

**Keywords:** bio-based, coating, organosilane, durability, polyurethane

## Abstract

Bio-based polyurethane (PU) coatings offer sustainable alternatives to petrochemical coatings but often suffer from inferior mechanical performance, durability, and chemical resistance. This work addresses that challenge by integrating a trifunctional bio-based crosslinker (glycerol) and a silane-based additive (hexamethyldisilane (HMDS)) to simultaneously enhance structural robustness and hydrophobicity. Coatings were synthesized using a renewable soybean oil polyol (SOP), glycerol (5, 10, 15 and 20 wt.%), and methylene diphenyl diisocyanate (MDI), followed by the addition of HMDS (10, 20, 30, 40 and 50 wt.%). Mechanical tests identified 10 wt.% glycerol as the optimal content, yielding a maximum tensile strength of 47.18 MPa. Incorporating 10 wt.% HMDS into the optimized formulation greatly increased water contact angle (WCA, 95.76°) and chemical resistance with minimal loss of mechanical performance (38.19 MPa, tensile strength); higher HMDS loadings caused network disruption and reduced strength. Calorimetry and thermogravimetric analyses confirmed that the modified coatings retained high thermal stability. This synergistic crosslinker additive strategy produced a structurally robust, water-resistant bio-based coating, demonstrating a viable high-performance sustainable coating solution for industrial applications.

## 1. Introduction

**Figure 1:**
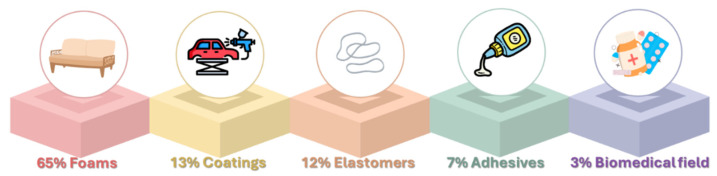
PU market. Adapted with permission from Ref. [3]. Copyright 2025 American Chemical Society.

Sustainable high-performance PU coatings, which are 13% shareholders of the PU market, are drawing intense interest as eco-friendly alternatives to petrochemical coatings in applications ranging from protective wood finishes to anti-corrosion metal primers (Figure 1) [1,2,3]. Conventional PU resins are prized for their durability, flexibility, and chemical resistance, making them a dominant choice for protecting wood and metal [4]. However, the environmental imperative to replace petroleum-derived polyols with renewable resources has spurred the development of bio-based PUs derived from vegetable oils and other biomass [5]. Notably, vegetable oil-based PU coatings have demonstrated excellent toughness, abrasion resistance, corrosion protection, and chemical resistance, all while reducing volatile organic compound emissions [6,7]. In particular, soybean oil (SO) an abundant, domestically available feedstock, can be converted into polyols for PU synthesis, providing a sustainable route to high-solid coatings and mitigating reliance on non-renewable petro-polyols [8,9]. Harnessing such bio-based polyols in PU coatings addresses both performance and sustainability, aligning with the demand for “greener” coatings without sacrificing the robust protection expected of industrial finishes [10,11,12].

Despite these advantages, bio-based PU coatings face key challenges like balancing mechanical robustness with surface durability (hydrophobicity, chemical/ink repellency), which is difficult due to trade-offs inherent in polymer design. Many vegetable-oil PUs tend to have lower thermal stability than petrochemical analogues and can exhibit reduced hardness if the bio-based polyol functionality is low. Simply increasing cross-link density or introducing rigid segments can improve hardness and solvent resistance, but often at the expense of flexibility and toughness, leading to brittle coatings prone to cracking. For example, hyperbranched or highly cross-linked bio-PUs show reinforced rigidity alongside markedly decreased elongation at break [13,14]. Conversely, reducing crosslinking or using flexible bio-polyols enhances coating ductility but undermines strength and barrier properties. Achieving simultaneously high mechanical strength and elasticity in bio-based PUs thus requires careful architectural control [15]. On the surface performance front, endowing PU coatings with extreme water/ink repellency and chemical resistance usually involves incorporating low-surface-energy components like fluoropolymers or silicones. Fluorinated additives are effective but raise environmental and toxicity concerns, motivating fluorine-free approaches. Silicone-based modifiers such as polydimethylsiloxane (PDMS) can dramatically lower surface energy and improve water resistance, yet their poor compatibility with the PU matrix often causes phase-separated domains that weaken mechanical integrity and transparency. Indeed, high PDMS loadings have been shown to reduce water absorption of a PU coating to ~1–2% but at the cost of halving the tensile strength (e.g., 15% PDMS lowered water uptake from 13.5% to 1.5% while tensile strength fell from 39 MPa to 21 MPa) [16]. These examples underscore the difficulty of simultaneously optimizing bulk mechanical properties and surface durability in bio-based PU systems—increasing one often compromises the other. New strategies are needed to break this compromise, enabling renewable PU coatings that are both mechanically robust and resistant to moisture, chemicals, and inks.

Recent research has explored multiple avenues to address this challenge. On the materials side, SOP has emerged as a promising base for sustainable PUs, owing to its triglyceride structure that can be chemically modified to introduce hydroxyl (-OH) functionality [17]. Epoxidation and ring-opening of SO, for instance, yields polyols with tailored -OH numbers and functionalities. The -OH functionality of a polyol strongly correlates with the cross-link density and glass transition of the resulting PU, and higher-functionality SOP has been shown to produce coatings with greater tensile strength, modulus, and toughness. In practice, researchers often generate SOP via glycerolysis (alcoholysis) by reacting epoxidized soybean oil (ESO) with polyhydric alcohols like glycerol to boost the -OH content. Hussain et al. exemplified this approach by synthesizing SOP through glycerol alcoholysis of ESO; the resulting PU coatings (60% bio-based content) exhibited increasing gel content (GC) and hardness as the isocyanate cross-linker ratio was raised, indicating successful network formation and improved coating rigidity [18]. Glycerol—a trifunctional, bio-derived polyol—is particularly attractive as a reactive crosslinker because it can multiply the cross-link density of vegetable oil-based polymers. Transesterification of triglycerides with glycerol yields mixtures of mono- and diglycerides with residual -OH groups, effectively creating polyols of higher average functionality that impart greater network connectivity in cured PUs. For example, monoglyceride routes (alcoholysis) have been widely used to produce alkyd-type SOP for two-component PU coatings, resulting in high-solid formulations (~60% bio content) with strong adhesion and enhanced hardness due to the increased cross-link density. Similarly, incorporating glycerol as a chain extender in hyperbranched oil-based PUs was reported to significantly improve coating hardness while maintaining excellent adhesion and flexibility. These studies demonstrate that judicious use of glycerol or other multi-OH bio-polyols can bolster the mechanical performance of bio-based PUs by creating a tightly cross-linked polymer network [13].

Complementary to backbone modifications, researchers have pursued silane and silicone-based additives to endow bio-PU coatings with hydrophobic, chemically durable surfaces. Silane coupling agents (e.g., trialkoxysilanes) and silicone polymers offer a route to introduce inorganic–organic hybrid structures into PUs. When incorporated into the PU matrix, organosilane molecules can hydrolyze and condense to form a polysilsesquioxane network interpenetrating the polymer, thereby elevating cross-linking and imparting superb water and weather resistance. For instance, PUs containing pendant silicone alkyl groups or siloxane segments exhibit significantly enhanced water repellency and outdoor weathering stability, along with improved adhesion to substrates, due to the formation of Si–O–Si networks that fortify the coating and bond with -OH-rich surfaces. Meng et al. recently demonstrated this concept by synthesizing an organosilicon castor-oil-based PU using a mercaptopropyl trimethoxysilane-modified isocyanate. The incorporation of silane side-groups in the hard segment led to Si–O–Si crosslinks upon curing, which more than doubled the tensile strength (from 9.5 MPa to 22.3 MPa) and raised the Young’s modulus nearly twenty-fold [15]. Although some elongation was sacrificed, the glass transition temperature (T_g_) and thermal stability of the coating increased, and its transparency, hydrophobicity, and chemical resistance were all remarkably improved with higher silane content. These results echo earlier findings that grafting silane onto PU backbones (for example, using 3-aminopropyltrimethoxysilane as a chain extender) can simultaneously enhance the mechanical strength, heat resistance, and water resistance of PU materials. In a related approach, Zhou et al. prepared a castor oil-based PU by blending in a -OH-terminated PDMS; the modified coating achieved a high pencil hardness of 6H alongside outstanding antifouling and anticorrosion performance on par with synthetic coatings [19]. These studies underscore that silicone/organosilane modifications are powerful tools for elevating the surface durability of bio-based PUs, providing hydrophobic, chemically resistant, and even self-cleaning surfaces, but the challenge is to integrate these benefits without compromising the bulk mechanical integrity [20].

From the above literature, a clear research gap emerges at the intersection of mechanical optimization and surface functionalization in sustainable PU coatings. Prior works have either concentrated on improving the polymer network strength (e.g., via hyperbranching, glycerol cross-linkers, or nano-fillers) or on enhancing surface properties (e.g., via fluorinated additives, PDMS segments, or silane coupling), but rarely in tandem. For example, a recent fluorine-free “antismudge” coating derived from SO and diphenylsilanediol (DPSD) demonstrated excellent water repellency and even the ability to resist permanent marker ink on wood, validating the concept of a bio-based, silicon-enhanced PU surface. However, that system relied on intrinsic network formation from the SOP and did not leverage small multifunctional crosslinkers like glycerol to maximize the polymer’s cross-link density. To the best of our knowledge, no study has yet combined a highly cross-linked bio-based PU network (through a glycerol-augmented SOP) with a post-polymerization silane treatment to reinforce surface durability. Achieving this combination would mean a single coating system that offers high mechanical robustness in its bulk and a low-surface-energy, damage-resistant skin, all derived from sustainable components [15].

The gap is addressed herein through the development of a novel bio-based PU coating system derived from SOP, with dual enhancements implemented to improve both the bulk and surface properties. First, glycerol is utilized as a crosslinking co-polyol to significantly increase the –OH functionality of the SOP, yielding a tightly cross-linked PU network for high mechanical strength and thermal stability. Second, after film formation, the coating surface is further modified with HMDS, a hydrophobic silane reagent, to create a thin silicon-containing passivation layer chemically bound to the PU surface. The HMDS treatment endows the coating with an extremely low surface energy and enhanced moisture/ink repellency, without introducing any fluorinated species. By integrating these strategies, the resulting SOP-, glycerol- and HMDS-based PU coatings exhibit exceptional hardness and cohesion in tandem with outstanding surface durability (high WCA, resistance to ink staining, and chemical inertness). It is demonstrated that the coatings can withstand rigorous mechanical wear and cyclic ink writing–erasing on wood, while also providing enduring corrosion protection on metal substrates, all while being predominantly bio-derived. To conduct a comprehensive study on the creation of structurally strong PU coatings with improved performance characteristics by modifying the bio-based composition with a glycerol cross-linker and an HMDS additive. The following sections detail the synthesis of the SOP, the formulation of glycerol-crosslinked PU films, HMDS-crosslinked PU films, and comprehensive characterization of the coating’s mechanical, thermal, chemical, and anti-smudge performance. Our findings highlight the potential of bio-based PUs to meet or exceed the stringent requirements of advanced coating applications, bridging the gap between sustainability and high-end functionality.

## 2. Experimental Section

### 2.1. Materials

SO was purchased from a local Walmart in Pittsburg, KS, USA. Glacial acetic acid (99.7%), hydrogen peroxide (H_2_O_2_) (29%), toluene (99.5%), amberlite IR 120H, sodium chloride (NaCl), sodium sulfate (Na_2_SO_4_), tetrafluoro boric acid (HBF_4_) (48 wt.%), lewitt MP64 (99.8%), and methanol (CH_3_OH) (99.9%), glycerol, HMDS were purchased from Fisher Scientific (Allentown, PA, USA). MDI was given by Huntsman (The Woodlands, TX, USA). BYK-054 (degassing agent) was purchased from Wallingford, CT, USA.

### 2.2. Synthesis of Soybean Oil Polyol

**Figure 2:**
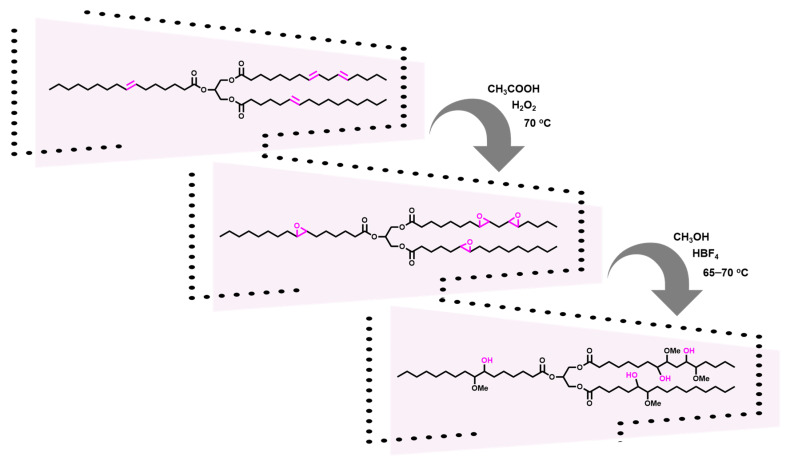
Synthesis of SOP in a two-step reaction.

SOP was synthesized through a two-step chemical modification process that involved epoxidizing the unsaturated fatty acid chains in SO, followed by the ring-opening of the oxirane groups (Figure 2). In the first step, a three-neck round-bottom flask equipped with a mechanical stirrer and temperature control held 500 g of SO, 250 mL of toluene, and 125 g of Amberlite IR 120H resin. The mixture was stirred for 15 min at ambient conditions to ensure homogeneity. Then, 78 mL of glacial acetic acid was added dropwise using a dropping funnel while keeping the reaction temperature below 10 °C with the aid of an ice bath. After completely adding the acetic acid (CH_3_COOH), the mixture was stirred for another 30 min. Following this, 442 mL of 30 wt.% H_2_O_2_ was added gradually in the same manner, and the reaction temperature was raised to 70 °C. The reaction continued under constant stirring for 7 h to ensure complete epoxidation. Once complete, the mixture was filtered to eliminate the ion-exchange resin. The organic phase was separated using brine solution (Saturated NaCl solution) and then dried over anhydrous Na_2_SO_4_. Finally, the solvent was removed by rotary evaporation to yield the ESO. The epoxy oxirane conversion percentage (EOC %) is calculated using Equation (S1).

In the second Step, a ring-opening reaction of ESO was performed using a 7:1 molar ratio of CH_3_OH to ESO. CH_3_OH and a catalytic amount of HBF_4_ were introduced into a round-bottom flask and stirred at 65–70 °C. After 1 h of pre-reaction mixing, ESO was added dropwise into the flask under constant stirring. Upon completion of the addition, the reaction mixture was cooled to room temperature, and Lewatit MP64 resin was introduced as a neutralizing agent. The suspension was stirred for an additional 45 min, followed by filtration to remove the resin. The final SOP product was obtained after solvent removal using rotary evaporation [21,22]. The characteristic data for SO, ESO and SOP are given in Table S1.

### 2.3. Synthesis of Polyurethane Films

#### 2.3.1. Synthesis of Glycerol-Based Films (GLY-X wt.%; X = 5, 10, 15 and 20)

A series of bio-based PU films were synthesized using SOP, MDI, and glycerol as a trifunctional crosslinking agent. The objective of incorporating different glycerol contents was to determine the optimal weight ratio for achieving the highest mechanical strength in the resulting PU films. Five formulations were prepared: a control film (CT), composed solely of SOP and MDI, and four experimental films containing glycerol at varying weight percentages of 5 wt.%, 10 wt.%, 15 wt.%, and 20 wt.% relative to the mass of SOP. These formulations were designated as GLY-5 wt.%, GLY-10 wt.%, GLY-15 wt.%, and GLY-20 wt.%, respectively, as shown in Table S2. In a typical synthesis, SOP and the corresponding amount of glycerol were introduced into a round-bottom flask and subjected to rotary evaporation at 60 °C for 30 min to remove residual moisture. After drying, the mixture was cooled to room temperature, and a stoichiometrically appropriate amount of MDI was added. The contents were further mixed using rotary evaporation at ambient conditions until a homogeneous viscous mixture was obtained. To eliminate entrapped air, a degassing agent (1 wt.% of the total mixture mass) was added to each formulation. The resulting mixture was then cast into Teflon-lined Petri dishes and allowed to cure at room temperature for 24 h. The CT film was synthesized using 5 g of SOP and MDI only, while the remaining formulations followed the same protocol with their respective glycerol concentrations.

#### 2.3.2. Synthesis of HMDS-Based Films (G-HMDS-X wt.%; X = 10, 20, 30, 40 and 50)

**Figure 3:**
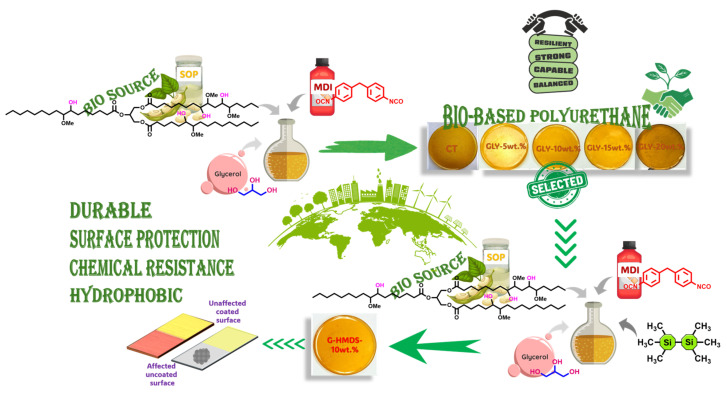
Synthesis process of PU films.

To further enhance the coating properties of the PU matrix, a series of HMDS-modified PU films were synthesized using the optimized formulation from the 10 wt.% glycerol-based PU film (GLY-10 wt.%), which demonstrated the highest mechanical strength among earlier formulations. Each HMDS-modified film contained a consistent composition of SOP (5.00 g), glycerol (0.50 g), and MDI (4.48 g). HMDS was incorporated as an additive at various weight percentages (10, 20, 30, 40, and 50 wt.%) relative to the total weight of the PU matrix (i.e., SOP + glycerol + MDI), as detailed in Table S3. In a typical procedure, SOP and glycerol were added to a round-bottom flask and dehydrated using rotary evaporation at 60 °C for 30 min. The mixture was then cooled to room temperature, after which MDI was introduced into the same flask. The contents were homogenized using rotary evaporation until a uniform prepolymer mixture was achieved. Subsequently, a degassing agent (1 wt.% relative to the combined weight of SOP, glycerol, and MDI) was added to remove any entrapped air. After degassing, the calculated amount of HMDS (as shown in Table S3) was incorporated into the mixture and stirred vigorously until a homogeneous and transparent blend was obtained. The final formulations were poured into Teflon-lined Petri dishes and allowed to be cured under ambient conditions (25 °C) for 24 h, resulting in five distinct HMDS-modified PU films with HMDS loadings of 10, 20, 30, 40, and 50 wt.%, which are formulated as G-HMDS-10 wt.%, G-HMDS-20 wt.%, G-HMDS-30 wt.%, G-HMDS-40 wt.% and G-HMDS-50 wt.%, respectively. Following the synthesis of the PU films, three representative samples, which are CT, GLY-10 wt.%, and G-HMDS-10 wt.%, were selected for viscosity measurements to evaluate the effect of compositional modifications on the rheological properties. The CT sample exhibited the highest viscosity at 2.6 Pa.s, followed by GLY-10 wt.% at 2.2 Pa.s, and the G-HMDS-10 wt.% sample showed the lowest viscosity at 1.7 Pa.s (Table S4). This decreasing trend in viscosity can be attributed to the molecular interactions and structural modifications induced by the additives. The reduction in viscosity for the GLY-10 wt.% sample, relative to the CT, is likely due to the incorporation of glycerol, which, despite increasing crosslink density, introduces a more flexible and branched network that enhances flowability under shear. The further decline in viscosity observed in the G-HMDS-10 wt.% sample may result from the hydrophobic and bulky nature of the HMDS moieties, which disrupt intermolecular hydrogen bonding and reduce chain entanglement within the PU matrix. Consequently, this leads to lower internal resistance to flow, thereby decreasing the overall viscosity of the material. The overall synthesis process of PU films is represented in Figure 3.

#### 2.3.3. Mechanism of Synthesized PU Materials

The formation of PU films in this study is based on the polyaddition reaction between -OH-terminated polyols and diisocyanates [23]. This process yields urethane linkages without generating by-products [24,25]. In the first series of films, SOP, prepared through epoxidation followed by ring-opening, serves as the primary polyol component. The -OH groups introduced during the ring-opening of the ESO react with MDI, a commonly used aromatic diisocyanate, to form urethane bonds. This reaction occurs readily under mild conditions, resulting in the formation of a linear or lightly branched polymer network, depending on the functionality of the polyols involved. To modify and enhance the network architecture and mechanical strength of the PU matrix, glycerol, a trifunctional alcohol, has been introduced as a crosslinking agent. Glycerol contains three -OH groups, enabling it to form a higher density of crosslinks compared to SOP, which possesses secondary -OHs. During the reaction, each -OH group of glycerol can react with an isocyanate (-NCO) group from MDI, resulting in a three-dimensional crosslinked structure. The incorporation of glycerol was systematically varied (5–20 wt.% concerning SOP) to optimize the mechanical properties of the PU films. It was found that the formulation containing 10 wt.% glycerol provided the best mechanical performance, likely due to an optimal balance between crosslinking density and segmental flexibility. At lower glycerol contents, the network may be too loosely crosslinked to resist deformation, while at higher concentrations, excessive crosslinking may lead to brittleness or phase separation.

Building upon the optimized 10 wt.% glycerol formulation, a second series of PU films was synthesized by incorporating HMDS as a performance-enhancing additive. HMDS, a silicon-containing organosilane, was added at varying concentrations (10–50 wt.% relative to the total glycerol-based PU matrix (GLY-10 wt.%) to tailor the surface and barrier properties of the films. Although HMDS lacks reactive functional groups that participate directly in the urethane-forming reaction, it can physically integrate into the polymer matrix through dispersion and weak intermolecular interactions. During the curing process, HMDS is believed to migrate toward the surface, resulting in a silicon-enriched layer that imparts hydrophobicity and thermal resistance to the PU films. The incorporation of HMDS into the PU matrix also influences the films’ microstructure. At moderate loadings, HMDS may create nanophase-separated domains within the PU network, potentially contributing to improved toughness and surface uniformity. However, excessive HMDS content could disrupt the matrix’s homogeneity, leading to phase incompatibility and reduced mechanical integrity. Thus, the interplay between chemical crosslinking via glycerol and physical modification via HMDS defines the dual-functionality design of the PU films developed in this study. Together, these molecular strategies provide a tunable platform for fabricating high-performance, bio-based PU coatings with enhanced mechanical strength, thermal stability, and surface functionality.

The wt.% of glycerol and HMDS were selected to provide a sufficiently distinct composition range for evaluating their effect on PU properties while avoiding excessive loading that could cause phase separation, over-crosslinking, or deterioration of the polymer network.

### 2.4. Characterizations

The ESO and its synthesized polyol derivative (SOP) were systematically characterized in accordance with relevant International Standards Organization (ISO) and American Society for Testing and Materials (ASTM) protocols to confirm their chemical structure, functional group conversion, thermal behavior, rheological properties, molecular weight evolution, and final coating performance. The iodine value is an important parameter used to determine the degree of unsaturation present in oils and their derivatives. It reflects the amount of carbon–carbon double bonds available in the material and is therefore useful for monitoring the conversion of SO during epoxidation. In this study, the iodine value of SO and the residual unsaturation in ESO were quantified using the Hanus method following IUPAC 2.205. The hydroxyl number is used to quantify the amount of hydroxyl groups present in a polyol and is one of the most important parameters for evaluating its suitability for PU synthesis, since hydroxyl groups directly participate in the urethane-forming reaction with isocyanates. In the present work, the -OH number was determined following the phthalic anhydride pyridine (PAP) method as per IUPAC 2.241. The acid value indicates the amount of free acidic species present in the sample and is useful for assessing the purity of the synthesized polyol as well as the extent of side reactions during synthesis. A lower acid value is generally desirable for PU preparation. Here, the acid value was assessed using the indicator titration method according to IUPAC 2.201. The oxirane oxygen content is used to evaluate the extent of epoxidation in vegetable oils by measuring the epoxy functionality introduced into the molecular structure. This parameter is particularly important for confirming the successful conversion of unsaturated double bonds into epoxide groups before further transformation into polyols. In this work, the oxirane oxygen content of ESO was evaluated using a titration method involving glacial acetic acid and tetraethylammonium bromide. Rheological analysis provides insight into the flow behavior and viscosity of liquid materials, which are critical for understanding processability and handling characteristics during synthesis and coating formation. Rheological measurements were performed using a TA Instruments AR 2000 ex rheometer (New Castle, DE, USA) equipped with a 2° cone plate of 12.5 mm radius. Fourier transform infrared (FTIR) spectroscopy is a widely used analytical technique for identifying functional groups based on their characteristic vibrational absorption bands. It is particularly useful for confirming the formation of epoxy groups, hydroxyl groups, and urethane linkages during the stepwise synthesis of PU materials. In this study, FTIR spectra were acquired using a PerkinElmer Spectrum spectrometer (Shelton, CT, USA), covering a spectral range of 4000 to 500 cm^−1^ to identify functional groups in the PU matrix. Proton nuclear magnetic resonance (^1^H NMR) spectroscopy provides detailed structural information by analyzing the chemical environment of hydrogen atoms in a molecule. This technique was used to verify the chemical structure of the synthesized intermediates and products and to confirm the successful transformation of SO into ESO and SOP. ^1^H NMR spectroscopy was carried out at ambient temperature using a Bruker AC-80 spectrometer operating at 400 MHz (Billerica, MA, USA). Deuterated chloroform (CDCl_3_) served as the solvent, and tetramethylsilane was used as the internal reference standard. Sample solutions were prepared at concentrations of 8 to 10 mg/mL in CDCl_3_. Gel permeation chromatography (GPC) is a size-exclusion technique used to determine the molecular weight distribution of polymeric materials and reaction products. It is especially helpful for monitoring molecular weight evolution during synthesis and for assessing whether chain extension or network development has occurred. In the present study, GPC was employed to monitor molecular weight evolution during the reaction, using a 150 mm long column with a 20 μm particle size. The column was eluted with tetrahydrofuran at a flow rate of 1 mL/min and maintained at 35 °C (Carlsbad, CA, USA). Thermogravimetric analysis (TGA) is used to examine the thermal stability and degradation behavior of materials by measuring weight loss as a function of temperature. It provides valuable information about decomposition stages and thermal resistance of PU films. In this work, TGA was conducted using a Discovery Series TGA 550 (TA Instruments, New Castle, DE, USA). Approximately 8 to 10 mg of sample was heated from 25 °C to 600 °C at a rate of 10 °C/min under a nitrogen flow of 40 mL/min in a platinum pan. The percentage weight loss versus temperature was plotted for thermal stability analysis. Differential scanning calorimetry (DSC) is a thermal analysis technique used to study heat flow associated with transitions such as glass transition, crystallization, and melting. For PU films, the T_g_ is an important parameter because it reflects chain mobility and network rigidity. DSC was performed using a TA Instruments Q100 DSC (New Castle, DE, USA) to determine the T_g_ of PU films. About 6 to 8 mg of each sample was sealed in an aluminum pan and subjected to heating and cooling cycles from −50 °C to 200 °C at a rate of 10 °C/min under a nitrogen atmosphere. Mechanical testing is essential for evaluating the strength and deformation behavior of polymer films under applied load. Tensile strength provides direct information about the load-bearing capability and structural integrity of the PU network. Mechanical testing was conducted using an Instron 3367 universal testing machine (Norwood, MA, USA) following ASTM D882-97 (Standard Test Method for Tensile Properties of Thin Plastic Sheeting, ASTM International, West Conshohocken, PA, USA, 15 August 2018) to determine the tensile strength of the PU films. The samples had dimensions of 10 mm width, 50 mm length, and 2.45 mm thickness, with tests performed at a crosshead speed of 10 mm/min. Results were averaged over three replicates. Hardness testing measures the resistance of a material surface to indentation and is commonly used to evaluate the rigidity and surface durability of polymer coatings and films. In this study, hardness measurements were carried out using a Type D Durometer (PTC Instruments, Los Angeles, CA, USA) according to ASTM D2240 standards (Standard Test Method for Rubber Property—Durometer Hardness, ASTM International, West Conshohocken, PA, USA, 23 July 2021). WCA measurement is a surface characterization technique used to assess the wettability and hydrophobicity of a material. A higher contact angle indicates lower surface energy and greater hydrophobic character, which are important for coating applications requiring moisture resistance and anti-smudge behavior. WCA measurements were performed using an Ossila Contact Angle Goniometer to evaluate the surface hydrophobicity of the prepared films.

## 3. Results and Discussion

### 3.1. Structural Characterization of Soybean Oil, Epoxidized Soybean Oil and Soybean Oil Polyol

SO, which naturally contains unsaturated double bonds, was chemically modified via a two-step reaction sequence. The first step involved epoxidation, wherein the carbon–carbon double bonds were converted into epoxy groups, yielding ESO. This was followed by a ring-opening reaction of the epoxide groups to produce -OH groups, resulting in SOP. The chemical transformations at each stage were confirmed using FTIR spectroscopy, ^1^H NMR spectroscopy, and GPC.

In the FTIR spectrum of SO, characteristic peaks at 1645 cm^−1^ and 3009 cm^−1^ were attributed to the stretching vibrations of the C=C bonds and the =C–H alkene bonds, respectively. These peaks disappeared in the ESO spectrum, confirming the successful epoxidation of the double bonds. The appearance of a new absorption band at 829 cm^−1^ indicated the presence of the epoxy group (C–O–C). Upon ring-opening of the epoxy groups, this peak disappeared in the SOP spectrum, and a broad absorption band emerged at 3462 cm^−1^, corresponding to O–H stretching vibrations, thereby confirming the formation of -OH groups (Figure S1a) [26]. The structural evolution of SOP was further verified by ^1^H NMR spectroscopy (Figure S1c) [27].

GPC separates and analyzes polymers in solution based on their hydrodynamic volume. As the polymer solution passes through a column packed with porous beads, larger molecules are excluded from entering the pores and elute earlier, while smaller molecules penetrate deeper into the pores and elute later. Due to the incorporation of -OH groups and an increase in molecular structure, it was anticipated that the synthesized SOP would exhibit a shorter retention time compared to ESO. This expectation is confirmed by the GPC chromatogram, where SOP elutes at 32.02 min, slightly earlier than ESO, which elutes at 32.30 min. Interestingly, SO elutes at 32.05 min, which is also earlier than ESO, contrary to typical expectations. This anomaly may be attributed to the presence of rigid oxirane rings in ESO, potentially inducing intramolecular folding or a more compact molecular conformation, thereby reducing its hydrodynamic volume and increasing its retention time relative to SO. Additionally, a minor shoulder observed at approximately 30.37 min may indicate the presence of dimeric species. These dimers likely arise from covalent or non-covalent interactions between two monomeric units, contributing to a distinct elution feature (Figure S1b) [21,28].

The significantly higher viscosity of SOP (2.38 Pa.s) compared to ESO (0.17 Pa.s) and SO (0.022 Pa.s) can be attributed to the increased polarity and enhanced intermolecular interactions introduced by the polyol structure. The ring-opening of epoxy groups during the functionalization process introduces -OH groups, resulting in increased molecular branching and a more complex, extended molecular architecture. These structural modifications enhance hydrogen bonding and other polar interactions, thereby increasing resistance to flow and contributing to the elevated viscosity of SOP (Table S1). In summary, the successful synthesis of SOP from SO via epoxidation and subsequent ring-opening was confirmed by FTIR, ^1^H NMR, and GPC analyses, demonstrating the formation of SOP suitable for further applications.

### 3.2. Fourier Transform Infrared Spectra of Polyurethane Films

**Figure 4:**
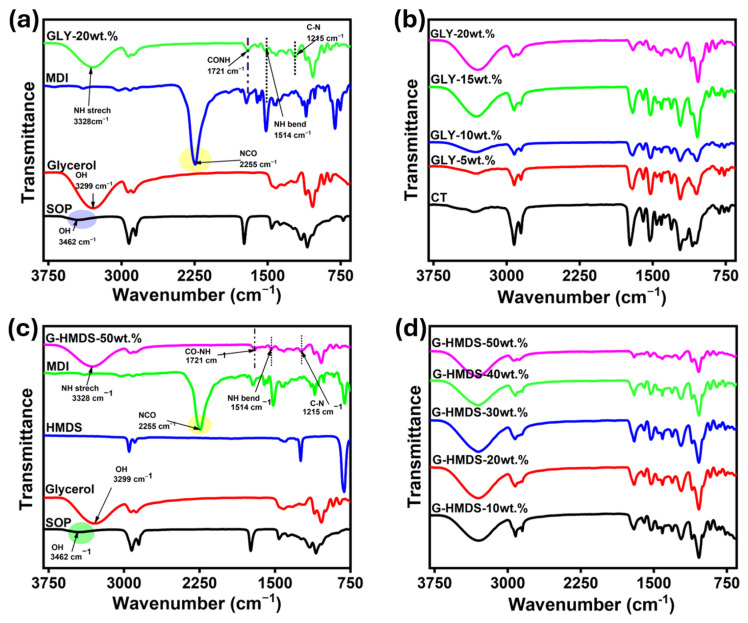
FTIR data. (**a**) Comparison of GLY-20 wt.% PU samples with its monomers; (**b**) comparison of different wt.% of glycerol; (**c**) comparison of G-HMDS-50 wt.% PU samples with its monomers; (**d**) comparison of different wt.% of HMDS.

For the preparation of PU material, the -OH groups of the polyol (SOP) and diol (glycerol) react with the -NCO groups present in diisocyanate (MDI). As shown in the FTIR spectra (Figure 4a), the characteristic –OH stretching vibrations of glycerol and SOP observed at 3299 cm^−1^ and 3462 cm^−1^, respectively, disappear in the spectrum of the synthesized PU material (GLY-20 wt.%), indicating the successful consumption of -OH groups during the reaction. Furthermore, the –NCO stretching vibration peak of MDI, initially observed at around 2255 cm^−1^, also disappears in the GLY-20 wt.% spectrum, confirming the effective incorporation of the -NCO groups into the polymer network. Additionally, the successful formation of PU is supported by the presence of characteristic urethane linkages in the FTIR spectrum of GLY-20 wt.%, including the carbonyl stretching vibration of the urethane (-CONH) at approximately 1721 cm^−1^, -NH bending at around 1514 cm^−1^, -CN stretching at 1215 cm^−1^, and -NH stretching vibration at 3328 cm^−1^. These features were consistently observed across all synthesized materials, confirming the successful formation of urethane linkages (Figure 4b) [29]. In conclusion, the disappearance of -OH and -NCO peaks and the emergence of characteristic urethane bands in the FTIR spectra provide strong evidence for the successful synthesis of PU materials from SOP, glycerol, and MDI.

After incorporating HMDS as an additive into the previously selected GLY-10 wt.% system to enhance the coating property of PU material, the spectra indicating urethane linkage remained the same, which indicates that the incorporation of HMDS did not affect the urethane formation of PU matrix (Figure 4c,d).

### 3.3. Tensile Strength of Polyurethane Films

**Figure 5:**
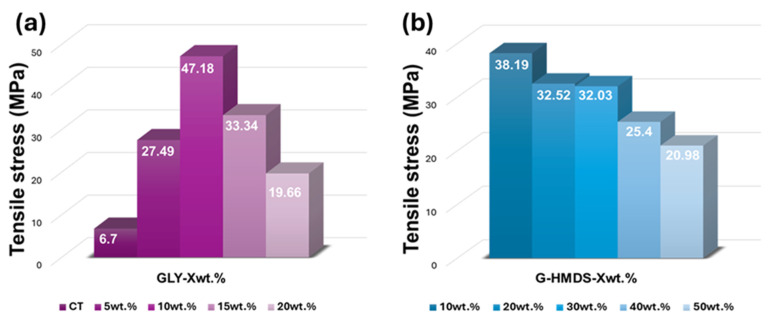
Tensile strength of (**a**) glycerol-based PU samples (GLY-X wt.%) and (**b**) HMDS-based PU samples (G-HMDS-X wt.%).

Mechanical strength is a crucial parameter for evaluating the performance of PU coatings, particularly in applications that require durability, structural stability, and long-term reliability. To assess the impact of glycerol content on the mechanical performance of the synthesized PU films, tensile strength measurements were conducted on the CT sample and PU films modified with 5 wt.%, 10 wt.%, 15 wt.%, and 20 wt.% glycerol, respectively. The results are summarized in Figure 5a and Table S4. The CT film, which included only SOP and MDI without any glycerol crosslinker, exhibited the lowest tensile strength of approximately 6.7 MPa, indicating a relatively weak and flexible polymeric network due to its lower crosslink density. Upon incorporating 5 wt.% glycerol, a significant increase in tensile strength to 27.49 MPa was observed. This enhancement results from the additional -OH functionalities introduced by glycerol, which participated in the formation of urethane bonds with MDI, thereby increasing the overall network density and intermolecular cohesion. The tensile strength significantly increased to 47.18 MPa with the addition of 10 wt.% glycerol, marking the peak mechanical performance among all the formulations tested. This optimized composition represents a critical balance between crosslinking density and segmental mobility. At this glycerol concentration, the network structure is sufficiently interconnected to withstand applied tensile stress while maintaining adequate chain flexibility for energy dissipation, resulting in a tough and resilient PU matrix. However, further increases in glycerol content to 15 wt.% and 20 wt.% led to a gradual decline in tensile strength to 33.34 MPa and 19.66 MPa, respectively. This decline is likely due to over-crosslinking and microphase separation effects, which may hinder the formation of a homogeneous polymeric matrix and induce internal stresses or brittleness. Excessive glycerol content can also result in the presence of unreacted -OH groups or irregular crosslink distribution, negatively impacting the film’s cohesive integrity under mechanical load. These findings highlight the significant role of controlled crosslinker incorporation in tuning the mechanical performance of bio-based PU coatings [30,31,32]. The 10 wt.% glycerol formulations not only achieve the highest tensile strength but also underscore the importance of optimizing the stoichiometric ratio of multifunctional polyols in network design [33]. The superior mechanical properties of this formulation establish it as a promising candidate for further functionalization, such as the subsequent incorporation of HMDS to tailor surface and barrier properties without compromising mechanical performance.

To develop robust and mechanically resilient PU coatings, the optimized formulation containing 10 wt.% glycerol (GLY-10 wt.%), previously found to deliver the highest tensile strength of 47.18 MPa among all glycerol-modified PU films, was chosen as the base system for further structural enhancement with HMDS. HMDS was incorporated in varying wt.% (10 wt.%, 20 wt.%, 30 wt.%, 40 wt.%, and 50 wt.%) relative to the total weight of the PU matrix to investigate its effect on mechanical properties and identify the optimal loading for potential coating applications [34,35]. As shown in Figure 5b and Table S4, adding 10 wt.% HMDS into the glycerol-crosslinked PU network (G-HMDS-10 wt.%) resulted in a tensile strength of 38.19 MPa, which, while slightly lower than the unmodified 10 wt.% glycerol system (GLY-10 wt.%), still represents a significant improvement over all other glycerol-only formulations (27.49 MPa for GLY-5 wt.%, 33.34 MPa for GLY-15 wt.%, and 19.66 MPa for GLY-20wt.%). This indicates that HMDS, at low concentrations, can integrate well within the crosslinked urethane matrix, likely enhancing microphase uniformity and providing a degree of stiffness through physical interaction and improved network packing density. As HMDS content increases, tensile strength gradually decreases to 32.52 MPa (G-HMDS-20 wt.%), 32.03 MPa (G-HMDS-30 wt.%), 25.40 MPa (G-HMDS-40 wt.%), and 20.98 MPa (G-HMDS-50 wt.%). This progressive reduction in tensile strength suggests that at higher concentrations, HMDS may lead to phase separation, poor dispersion, or reduced interfacial compatibility with the PU backbone. The high hydrophobicity and low reactivity of HMDS may also limit its ability to effectively reinforce the polymer network beyond a certain threshold, potentially leading to defects or weak zones under mechanical stress [36]. Notably, all HMDS-modified PU films demonstrated higher mechanical performance than the control PU film (6.7 MPa) and the GLY-20 wt.% (19.66 MPa) formulation, reinforcing the conclusion that the hybrid incorporation of silane-based additives is an effective strategy for improving strength in bio-based PU coatings. The 10 wt.% HMDS formulation (G-HMDS-10 wt.%) emerged as the most promising candidate, balancing tensile performance with compositional compatibility, and was therefore selected for subsequent surface coating evaluations. These findings illustrate the synergistic potential of combining controlled chemical crosslinking (via glycerol) with organosilane additives (HMDS) to fine-tune the structural and mechanical properties of sustainable PU materials, enabling their application in next-generation functional coatings. Figure S2 represents the tensile strength with error bars for glycerol-based and HMDS-based PU samples.

### 3.4. Hardness Test of Polyurethane Films

Hardness is a crucial mechanical property for PU coatings, directly influencing their resistance to surface deformation, abrasion, and wear under mechanical or environmental stress. In protective and structural coating applications, higher surface hardness typically results in enhanced durability, scratch resistance, and extended material lifespan. Thus, optimizing the hardness of bio-based PU films is essential for ensuring their viability in real-world functional environments.

**Figure 6:**
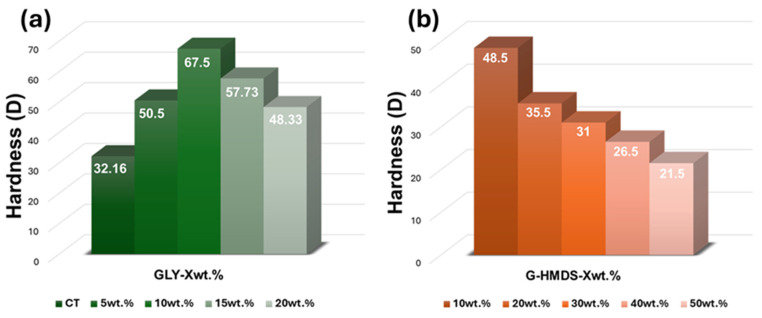
Hardness strength of (**a**) glycerol-based PU samples (GLY-X wt.%) and (**b**) HMDS-based PU samples (G-HMDS-X wt.%).

To evaluate the influence of glycerol content on hardness, Shore D hardness measurements were conducted on the CT film and on PU films modified with varying glycerol contents (5, 10, 15, and 20 wt.%). As shown in Figure 6a and Table S4, the control film, composed solely of SOP and MDI, exhibited a relatively low hardness of 32.16, indicative of a soft and under-crosslinked polymeric structure. This baseline reflects the flexible and elastomeric nature of the network formed exclusively by SOP’s secondary -OH and MDI, resulting in limited chain rigidity and surface resistance. Upon adding 5 wt.% glycerol (GLY-5 wt.%), hardness significantly increased to 50.5, demonstrating the effect of enhanced crosslinking density through the introduction of additional primary -OH groups. This trend continued with GLY-10 wt.%, where the hardness peaked at 67.5, suggesting optimal structural packing and segmental restriction within the polymer network. The tri-functional nature of glycerol promotes the development of a more densely crosslinked and rigid matrix, which leads to superior resistance against indentation or surface penetration. However, further increases in glycerol content beyond 10 wt.% resulted in decreased hardness, with values dropping to 57.73 for GLY-15 wt.% and 48.33 for GLY-20 wt.%. This reduction indicates that excessive glycerol may lead to phase inhomogeneity, over-crosslinking, or microdomain formation that undermines the uniformity of the film’s surface. Overloading with highly functionalized polyols can also introduce internal stresses and disrupt the optimal packing of the polymer chains, resulting in slightly softer and less structurally stable films. Overall, the results suggest that 10 wt.% glycerol represents the ideal crosslinker concentration for maximizing surface hardness in the synthesized bio-based PU films. This balance ensures adequate crosslink density for rigidity while avoiding defects or phase separation associated with higher glycerol content. The enhancement in hardness, along with improvements in mechanical strength, further supports the choice of this formulation for downstream applications in high-performance coating systems.

Shore D hardness testing was conducted on HMDS-modified PU films synthesized using a 10 wt.% glycerol-based formulation as the base matrix to evaluate the impact of HMDS incorporation on surface rigidity and durability. As shown in Figure 6b and Table S4, the results indicate that while HMDS affects hardness behavior, its inclusion results in a decrease in surface hardness compared to the unmodified glycerol-based PU film (GLY-10 wt.%), which had the highest recorded hardness of 67.5. The G-HMDS-10 wt.% film demonstrated a Shore D hardness of 48.5, which, although still significantly higher than the control film (CT, 32.16), is notably lower than the optimized GLY-10 wt.%. This reduction suggests that while HMDS may enhance thermal and hydrophobic properties, it does not strengthen surface rigidity to the same extent as the chemical crosslinking induced by glycerol. Since HMDS is a non-reactive organosilane that does not chemically bond with the PU matrix, its reinforcement mechanism is primarily physical. At low concentrations, HMDS may facilitate moderate structural densification; however, it lacks the molecular anchoring that -OH-bearing polyols provide for urethane linkage formation, thus limiting its ability to enhance hardness. As HMDS loading increased beyond 10 wt.%, a consistent decline in Shore D hardness was observed: 35.5 for G-HMDS-20 wt.%, 31.0 for G-HMDS-30 wt.%, 26.5 for G-HMDS-40 wt.%, and 21.5 for G-HMDS-50 wt.%. This trend indicates that excessive incorporation of HMDS may lead to microphase separation, poor dispersion, and soft domain formation within the PU matrix. These domains likely act as internal plasticizers, reducing crosslink integrity and diminishing the film’s resistance to surface indentation [37]. Overall, while HMDS does contribute to hardness improvements over the CT film, its presence does not exceed the reinforcement provided by optimal glycerol crosslinking. The results highlight the importance of balancing chemical crosslinking density with additive dispersion in designing multifunctional PU systems. In the context of this study, the GLY-10 wt.% formulation remains the most structurally rigid system, while G-HMDS-10 wt.% film offers a compromise between mechanical integrity and potential surface functionalization. Table 1 summarizes the results from previously reported bio-based PU materials.

### 3.5. Thermogravimetric Analysis and Derivative Thermograms of Polyurethane Films

**Figure 7:**
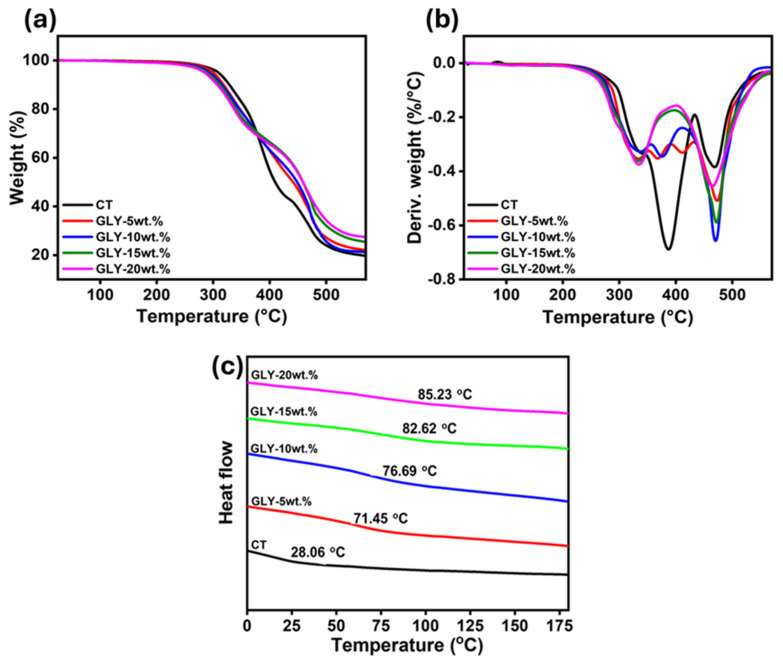
(**a**) TGA, (**b**) DTGA, and (**c**) DSC spectra of glycerol-based PU samples (GLY-X wt.%).

TGA and derivative thermogram (DTGA) were conducted under a nitrogen atmosphere to assess the thermal stability of the synthesized PU materials. These analytical techniques offer critical insights into the thermal degradation behavior and structural stability of the materials’ key parameters for determining their suitability in various applications. While TGA curves illustrate the percentage of mass loss as a function of temperature, DTGA curves provide detailed information about the specific degradation phases. The primary objective of this analysis was to evaluate the effect of incorporating glycerol at various weight percentages, as well as the impact of adding 10 wt.% of HMDS, on the thermal characteristics of the PU systems, particularly in terms of their thermal stability. The TGA curves, depicted in Figure 7a, reveal that the CT sample exhibits a delayed onset of thermal degradation compared to all glycerol-modified formulations, as supported by the data summarized in the corresponding Table 2. The amount of solid residue remaining after thermal decomposition serves as a critical indicator of thermal stability, with higher char residue values reflecting greater resistance to complete breakdown. A stable char layer can serve as a protective thermal barrier, helping to prevent further degradation of the underlying polymer. Notably, reduced thermal stability does not necessarily imply an earlier onset of degradation. In fact, the inclusion of glycerol may enhance the formation of a thermally stable char layer that becomes increasingly effective at elevated temperatures. In terms of char residue content, a clear increasing trend is observed with rising glycerol concentration in the PU matrix [52,53]. The specific char residue percentages are as follows: 18.52% for the CT sample, 21.00% for GLY-5 wt.%, 20.90% for GLY-10 wt.%, 24.70% for GLY-15 wt.%, and 26.66% for GLY-20 wt.%. The CT formulation, exhibiting the lowest char residue, is consequently the least thermally stable among all examined samples. Conversely, the steady increase in char residue with higher glycerol loadings suggests that glycerol contributes to improved thermal robustness of the PU materials. In Figure 7b, DTGA spectra further elucidate the degradation pattern, showing a two-step thermal degradation occurring between 300 °C and 500 °C for all glycerol-based PU samples. The first degradation event, located in the 300–400 °C range, corresponds to the decomposition of the PU matrix, while the second degradation observed above 400 °C is attributed to the degradation of the previously formed char residues. The enhanced thermal stability resulting from glycerol incorporation is further evidenced by the shift in T_MAX_ (temperature at the maximum weight loss rate) to higher values in all glycerol-containing samples relative to the CT sample, as documented in Table 2. This upward shift in T_MAX_ underscores the stabilizing influence of glycerol on the thermal degradation behavior of the PU network. Moving on to the TGA and DTGA data for the G-HMDS-10 wt.% sample (Figure S3a), no notable deterioration in thermal behavior is detected upon HMDS incorporation. This is demonstrated by the retention of the same T_MAX_ value (470 °C), indicating that HMDS does not negatively impact the degradation kinetics of the PU system. Furthermore, a slight increase in char residue is recorded for the G-HMDS-10 wt.% sample (21.98%) compared to 20.9% for the GLY-10 wt.% sample, signifying a minor improvement in thermal stability due to HMDS addition. In conclusion, both glycerol and HMDS additives positively influence the thermal stability of the PU adhesives. Glycerol enhances char formation and raises degradation temperatures, while HMDS contributes to incremental thermal reinforcement without compromising structural integrity. These modifications underscore the potential of tailored PU formulations for high-performance, thermally demanding applications.

### 3.6. Differential Scanning Colorimetry of Polyurethane Films

DSC was employed to investigate the influence of glycerol content on the thermal transitions and molecular mobility of the synthesized PU films. The corresponding DSC thermograms for the CT and glycerol-modified PU films (containing 5, 10, 15, and 20 wt.% glycerol) are shown in Figure 7c. These thermograms provide valuable insight into the extent of segmental motion, crosslinking density, and thermal coherence within the polymer matrices. All PU films exhibited broad, continuous endothermic transitions without the presence of sharp peaks, which is an indication typical of amorphous and crosslinked polymer systems. The absence of distinct melting transitions confirms the predominantly non-crystalline nature of the PU materials, with their thermal response being governed primarily by segmental mobility rather than phase transitions associated with ordered domains. At room temperature, the PU materials exist in a glassy, rigid state, characterized by low segmental mobility. As the temperature approaches the T_g_, an increase in molecular mobility is reflected by a noticeable shift in the baseline of the heat flow curve. Beyond T_g_, the polymer enters a rubbery state, enhancing its flexibility and facilitating segmental chain movement. As illustrated in Figure 7c and summarized in the accompanying Table 2, the CT sample exhibited the lowest T_g_ at 28.06 °C. With increasing glycerol content from 5 wt.% to 20 wt.%, a consistent rise in T_g_ was observed. This trend is attributed to both structural and compositional changes introduced by glycerol in the PU network. Glycerol, a trifunctional alcohol containing three -OH groups, serves as a crosslinking agent during PU synthesis. As its concentration increases, the crosslink density within the polymer network correspondingly rises, resulting in a more rigid and restricted molecular architecture. This increased rigidity limits the mobility of polymer chains and contributes to the elevation of T_g_ [52]. These findings are further corroborated by mechanical testing results, including tensile strength (Figure 5) and hardness (Figure 6), which also reflect enhanced network rigidity. Furthermore, the presence of polar -OH groups from glycerol enhances intermolecular hydrogen bonding within the matrix, which additionally restricts molecular motion and further contributes to the increase in T_g_. Collectively, the observed rise in T_g_ with increasing glycerol content underscores the formation of a more highly crosslinked and thermally stable PU structure. In addition, analysis of the DSC spectra for PU samples containing HMDS as an additive (Figure S3b) indicates that the thermal stability of the PU system remains unaffected by HMDS incorporation. Importantly, a clear upward trend in T_g_ is observed as the HMDS content increases from 10 wt.% to 50 wt.%. This behavior can be attributed to the thermal enhancement imparted by silane-based compounds, such as HMDS, which are known to improve the thermal resistance and stiffness of PU systems. The inclusion of HMDS is likely to contribute to increased structural organization and restricted chain dynamics, thus promoting higher T_g_ values with increasing additive concentration. In conclusion, both glycerol and HMDS effectively enhance the T_g_ of PU films through increased crosslinking and restricted chain mobility. These findings highlight the potential of multifunctional alcohols and silane additives to tailor the thermal behavior of PU systems for advanced material applications.

### 3.7. Gel Content and Degree of Swelling of Polyurethane Films

**Figure 8:**
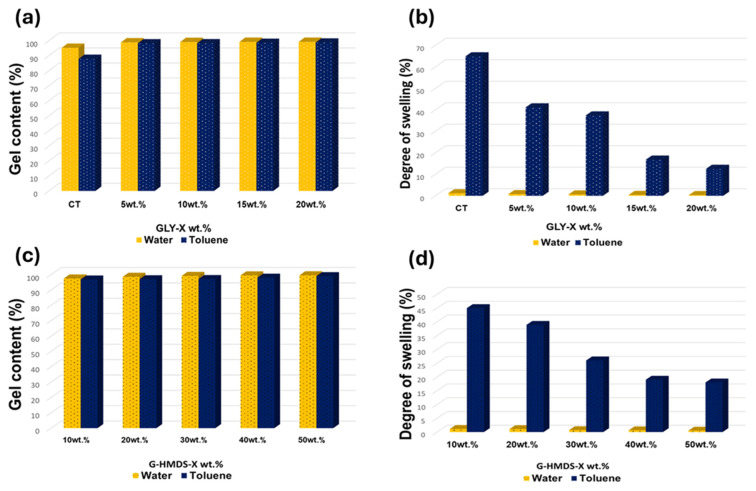
(**a**) GC of GLY-X wt.% PU samples; (**b**) DS of GLY-X wt.% PU samples; (**c**) GC of G-HMDS-X wt.% PU samples; (**d**) DS of G-HMDS-X wt.% PU samples.

GC analysis was conducted to assess the degree of crosslinking in the synthesized PU films based on glycerol and HMDS concentration. GC indicates the portion of the polymer network that remains insoluble in solvents, serving as a direct measure of crosslink density, network integrity, and chemical stability [54]. A higher GC signifies a more robust, tightly crosslinked network that resists solvent swelling and degradation. The GC was evaluated using two solvents with different polarities: water, which represents a highly polar medium, and toluene, which is a nonpolar organic solvent. This dual-solvent method allows for the assessment of both hydrophilic and hydrophobic network integrity. The GC can be calculated using Equation (S2). As shown in Figure 8a, the CT film, synthesized without glycerol, demonstrated GC values of 95.16% in water and 87.78% in toluene. The lower value in toluene suggests that the CT film contains a significant fraction of soluble, low molecular weight components or inadequately crosslinked regions that can be easily extracted by nonpolar solvents. After incorporating glycerol at 5 wt.%, a significant increase in GC was observed: 98.81% in water and 98.09% in toluene, indicating that glycerol effectively enhances crosslink formation through its trifunctional -OH structure. This increase implies the development of a more cohesive three-dimensional polymer network that resists solvent extraction. The GC continued to rise slightly with increasing glycerol concentration, reaching a near plateau at 99.05% (water) and 98.12% (toluene) for the GLY-10 wt.% formulation. Beyond this point, additional increases in glycerol content to 15 wt.% and 20 wt.% provided only marginal improvements, with GC values stabilizing around 99.15–99.16% in water and 98.41–98.63% in toluene. This plateauing behavior indicates that the system reaches a saturation point in terms of effective crosslinking; further glycerol beyond this threshold contributes minimally to network densification and may introduce slight phase irregularities or unreacted -OH groups. Notably, the consistently higher GC in water compared to toluene across all formulations reflects the relatively hydrophobic nature of the PU networks formed with glycerol and SOP. The slightly lower values in toluene may be attributed to the nonpolar solvent’s ability to penetrate and slightly swell areas of the polymer network with less polarity or weaker crosslinking. Overall, the GC analysis confirms that glycerol acts as a highly efficient crosslinking agent in the PU system, significantly enhancing the chemical resistance and network integrity of the films. The GLY-10 wt.% formulation emerges as the optimal balance point, exhibiting high GC in both solvents while previously demonstrating superior mechanical and thermal properties. These findings further validate its selection as the most promising candidate for durable and solvent-resistant bio-based PU coatings.

The degree of swelling (DS) provides critical insight into the crosslink density and solvent resistance of PU networks. It signifies the extent to which a polymer can absorb solvent without dissolving, and it is inversely related to the tightness of the crosslinked network. Higher DS values indicate looser structures with greater free volume and chain mobility, while lower DS values suggest a dense, tightly crosslinked matrix that resists solvent uptake. The DS can be calculated using Equation (S3). As shown in Figure 8b, the DS of glycerol-based PU films was evaluated in both water (polar) and toluene (nonpolar) solvents. The CT film, which lacks glycerol, exhibited the highest swelling in both media: 19% in water and 64.9% in toluene, highlighting its relatively low crosslink density and weak resistance to solvent penetration. The sharp contrast between water and toluene swelling reflects the hydrophobic nature of the PU network formed without glycerol, which swells more easily in nonpolar solvents like toluene. With the addition of 5 wt.% glycerol, DS decreased significantly to 0.79% in water and 41.15% in toluene, indicating improved crosslinking. This reduction continued with GLY-10 wt.% (0.64% in water, 37.4% in toluene), marking a consistent trend of decreased swelling as glycerol content increases. The presence of tri-functional glycerol enhances the formation of a more compact, three-dimensional polymer network, thus reducing solvent diffusivity and swelling capacity. A more dramatic drop in DS was observed in GLY-15 wt.% (0.43% in water, 16.84% in toluene) and further in GLY-20 wt.% (0.34% in water, 12.57% in toluene). These values suggest that at higher glycerol concentrations, the PU network becomes highly crosslinked and solvent-impermeable. This correlates with the GC analysis, which showed nearly complete insolubility across all glycerol-containing samples. Notably, swelling in toluene decreased more steeply than in water, indicating enhanced hydrophobic stabilization within the network, likely due to improved packing and reduced segmental mobility from the additional crosslinks. The relatively low swelling percentages in water across all samples (≤1.19%) indicate that even the CT formulation has some hydrophilic resistance, possibly attributed to the nature of the SOP. However, the clear trend of decreasing DS with increasing glycerol content strongly supports glycerol’s role as a crosslinking enhancer. These findings align with GC and mechanical property data, reinforcing that 10–15 wt.% glycerol provides an optimal range for achieving both structural rigidity and solvent resistance in bio-based PU coatings.

To further understand the structural integrity and solvent resistance of the PU network following HMDS modification, GC analysis was performed on the HMDS-incorporated films derived from the optimal 10 wt.% glycerol-based matrix (GLY-10 wt.%). As shown in Figure 8c, all HMDS-modified PU films exhibited high GC in both water and toluene, indicating the formation of chemically stable, highly crosslinked networks even after silane modification. The GC in water progressively increased from 97.5% at G-HMDS-10 wt.% loading to 99.6% at G-HMDS-50 wt.%, while the corresponding values in toluene ranged from 96.83% to 98.98%, respectively. This trend reveals two key insights. First, the increase in GC with higher HMDS concentration suggests that HMDS enhances physical reinforcement and matrix cohesion, despite its lack of chemical reactivity with isocyanates or polyols. Likely, HMDS is physically entrapped or finely dispersed within the polymer matrix during curing, enhancing network compaction and reducing the proportion of extractable segments. Second, the minimal difference between water and toluene GC values at higher HMDS loadings implies excellent solvent resistance in both polar and nonpolar media, an essential attribute for coatings that may be exposed to chemically aggressive environments. Interestingly, although HMDS is not expected to participate in chemical crosslinking, its inclusion seems to promote the physical stabilization of the polymer network. This could stem from HMDS-induced microstructural densification or possible weak secondary interactions (e.g., van der Waals or hydrogen bonding) that further restrict chain mobility and solvent penetration. The sharp improvement from 10 wt.% to 30 wt.% HMDS suggests an optimal window where physical entrapment and interphase interactions are most effective. Beyond 30 wt.%, the GC reaches a plateau (≥99.5% in water and ≥98.4% in toluene), indicating saturation of the reinforcing effect. The slightly lower values in toluene for all samples, especially at 10 wt.%, reflect the greater capacity of nonpolar solvents to penetrate and extract loosely bound or uncrosslinked components, which are progressively minimized as HMDS content increases. In conclusion, the GC analysis confirms that HMDS incorporation does not compromise the chemical stability of the PU network. Rather, it contributes to further densification and solvent resistance, making the modified PU films suitable for high-performance coatings. This complements earlier mechanical and thermal findings and supports the multifunctional enhancement potential of HMDS in glycerol-crosslinked PU systems.

In this study, the swelling behavior of HMDS-modified PU films derived from the optimized 10 wt.% glycerol-based formulation (GLY-10 wt.%) was assessed. Swelling was evaluated in both water and toluene, representing polar and nonpolar environments, respectively. As shown in Figure 8d, the control HMDS-modified PU film containing G-HMDS-10 wt.% exhibited a DS of 1.13% in water and 45% in toluene. The high DS in toluene at this concentration suggests that the PU matrix, although moderately crosslinked, still retains free volume or less compact regions that allow significant solvent diffusion. In contrast, the lower DS in water indicates a moderately hydrophilic network resulting from the SOP and glycerol backbone, which remains largely resistant to polar solvent uptake. With increasing HMDS content, DS values progressively declined in both solvents. In G-HMDS-20 wt.%, the DS dropped to 1.08% in water and 38.9% in toluene, followed by further reductions in G-HMDS-30 wt.% (0.72% in water, 26% in toluene), G-HMDS-40 wt.% (0.65% and 19%), and ultimately in G-HMDS-50 wt.%, where the values stabilized at 0.52% in water and 18.65% in toluene. This consistent decline confirms that increasing HMDS concentration contributes to reduced solvent uptake, likely due to enhanced microstructural densification and reduced chain mobility. Although HMDS does not chemically crosslink, it appears to act as a physical reinforcing agent, enhancing matrix packing and reducing free volume, thereby restricting solvent diffusion pathways. The more pronounced decrease in DS with respect to toluene, compared to water, underscores the improved resistance of the PU films to nonpolar solvent swelling. This behavior suggests that the HMDS molecules are not merely dispersed within the matrix but also effectively suppress hydrophobic interactions between the PU network and nonpolar solvents. This is particularly advantageous for coatings or films that require solvent barrier properties in chemically aggressive or oil-rich environments. Notably, the DS results closely align with GC observations, further validating that higher HMDS loadings result in more physically stable and solvent-resistant networks. While chemical crosslinking remains unchanged, the physical compactness introduced by HMDS significantly limits the swelling capacity, especially in toluene. The plateau in DS values between 40 and 50 wt.% HMDS also indicates a saturation effect, beyond which further additions do not substantially enhance swelling resistance. In summary, the incorporation of HMDS into the glycerol-crosslinked PU system significantly improves solvent resistance, particularly against nonpolar solvents. The 40 wt.% HMDS formulation represents a highly resistant matrix, suitable for applications demanding exceptional dimensional and chemical stability. The visual representation of the tested samples is shown in Figure S4.

### 3.8. Coating Tests of Polyurethane Films

#### 3.8.1. Evaluation of Coating Durability via Color Retention Test

**Figure 9:**
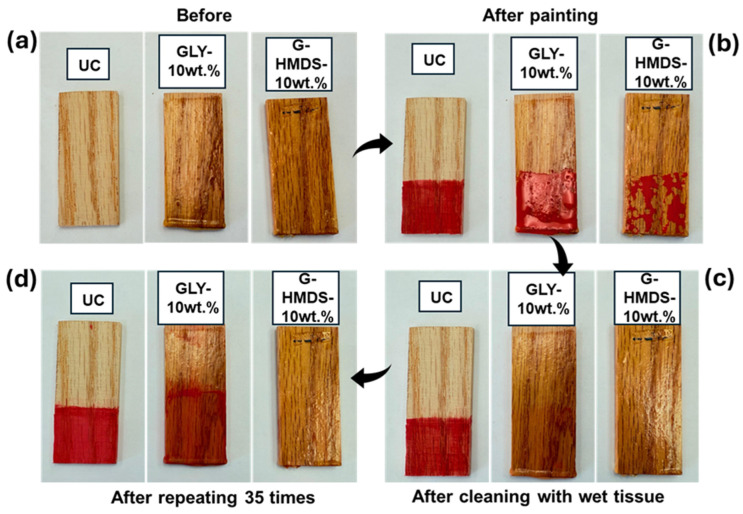
Visual demonstration of paint repellency and durability of untreated control (UC), GLY-10 wt.%, and G-HMDS-10 wt.% coated wood surfaces. (**a**) Surface appearance of samples before painting. (**b**) Paint application showing different wetting behaviors across samples. (**c**) After cleaning the painted surfaces with a wet tissue. (**d**) Paint removal performance after 35 cleaning cycles.

To evaluate the protective and self-cleaning properties of the synthesized PU coatings, a comparative color test was conducted on three different wood substrates: uncoated (UC), PU coated with 10 wt.% glycerol (GLY-10 wt.%), and PU coated with 10 wt.% glycerol plus 10 wt.% HMDS (G-HMDS-10 wt.%). The test aimed to simulate repeated exposure and surface cleaning under dye-staining conditions, thereby assessing the coatings’ resistance to pigment penetration and mechanical cleaning [55,56]. As shown in Figure 9a, all wood substrates were initially free of surface color. Each specimen was painted with red ink dye for 10 s (Figure 9b), followed by gentle wiping with a slightly moistened tissue to remove superficial dye. After the first exposure cycle (Figure 9c), the UC wood displayed immediate and irreversible staining, confirming its porous and hydrophilic surface nature. In contrast, the GLY-10 wt.% coated wood initially resisted dye adhesion and could be effectively cleaned with a wet tissue, indicating partial surface protection provided by the PU coating. Interestingly, the G-HMDS-10 wt.% coated sample exhibited superior hydrophobicity and stain resistance, with dye removal appearing notably more complete and uniform. To assess long-term performance, the immersion–wiping cycle was repeated for 35 consecutive cycles. After 35 cycles (Figure 9d), the GLY-10 wt.% coated specimen showed significant dye retention and visible color embedding into the coating, as evidenced by the reddish tint persisting even after cleaning. This suggests progressive degradation or weakening of the protective barrier, likely due to localized pigment penetration over repeated exposure. Conversely, the G-HMDS-10 wt.% coated specimen maintained its original surface appearance and remained largely free of dye uptake after 35 cycles (Figure 9d), even after cleaning with a wet tissue. This behavior clearly illustrates the enhanced stain repellency, surface durability, and hydrophobic coating performance imparted by the HMDS-modified PU network. These results directly support the functional advantage of HMDS incorporation into the PU matrix. The silane-modified coating significantly inhibits pigment adhesion and absorption, even under cyclic exposure and mechanical cleaning. The findings affirm the central objective of this study to enhance coating properties for long-term surface protection through the strategic incorporation of hydrophobic silane additives into a bio-based crosslinked PU system.

#### 3.8.2. Surface Protection Evaluation via Ink Stain Resistance Test

**Figure 10:**
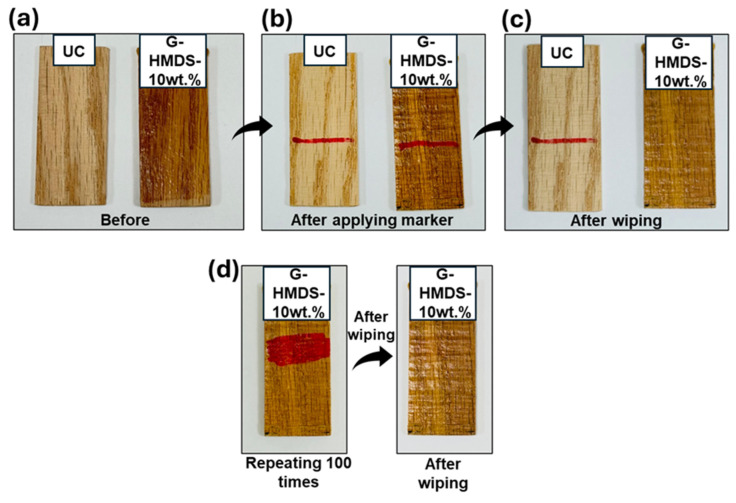
Marker ink repellency and reusability test of untreated control (UC) and G-HMDS-10 wt.% coated wood surfaces. (**a**) Initial appearance of both UC and G-HMDS-10 wt.% samples. (**b**) Application of a red marker line (**c**) after wiping with tissue and (**d**) after 100 repetitions of writing and wiping on the G-HMDS-10 wt.% sample.

To further assess the durability and stain-repellent properties of the HMDS-modified PU coatings, an ink resistance test was conducted on both UC and coated wood surfaces. The coated specimen consisted of a wood substrate treated with an optimized PU formulation of G-HMDS-10 wt.%. The experiment aimed to simulate the repetitive staining and cleaning conditions typically encountered in real-world applications [29]. As shown in Figure 10, both UC and coated wood panels were initially free from any ink markings (Figure 10a). A red ink line was applied to each sample using a permanent ink pen (Figure 10b), followed by gentle wiping with a slightly moistened tissue to evaluate stain removal (Figure 10c). Immediately after the first application, the UC surface absorbed the ink deeply, and the pigment remained clearly visible even after wiping, demonstrating the poor stain resistance of untreated wood due to its open pore structure and high surface energy. In contrast, the G-HMDS-10 wt.% coated surface initially resisted ink penetration effectively. After wiping, the ink was nearly completely removed, indicating a strong hydrophobic nature and low surface energy of the silane-modified PU layer. This behavior highlights the initial surface protection provided by the coating. To assess long-term durability, the ink application and wiping process were repeated for 100 cycles on the same coated surface. After 100 repetitions, the coated wood panel continued to exhibit excellent stain repellency and surface clarity, with no observable ink residue or discoloration, as seen in Figure 10d (bottom panels). This performance confirms the superior mechanical integrity and chemical resistance of the HMDS-containing PU network. The silane groups, while not chemically bonded to the polymer backbone, likely migrate to the surface during film formation, creating a protective hydrophobic layer that effectively repels both polar and nonpolar contaminants. These findings conclusively demonstrate that HMDS- functionalized PU coatings provide exceptional barrier performance and long-term durability, even under repetitive use and cleaning conditions. This result aligns well with prior GC, swelling resistance, and mechanical tests, further reinforcing the role of HMDS as a multifunctional additive that enhances both structural and surface protection properties in bio-based PU coatings [57].

#### 3.8.3. Chemical Resistance Test

**Figure 11:**
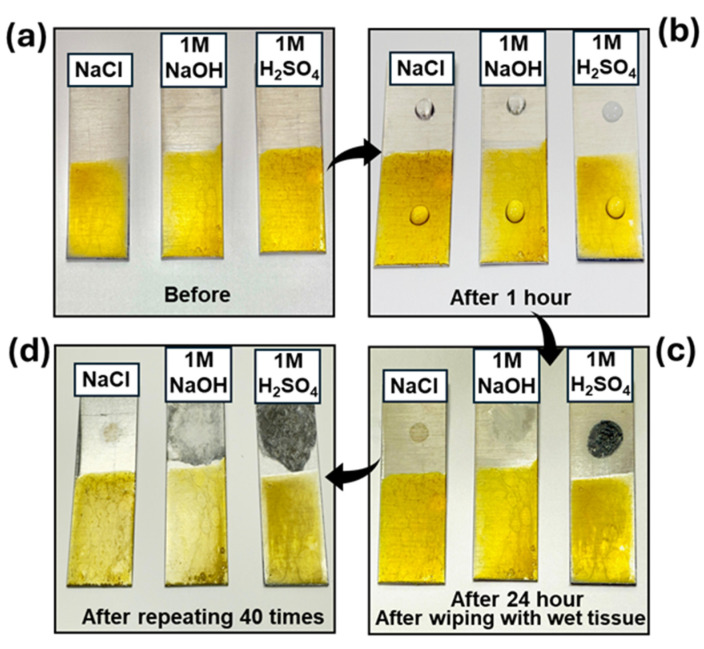
Evaluation of chemical durability and surface resistance of the modified coating against corrosive environments (NaCl, 1 M NaOH, and 1 M H_2_SO_4_). (**a**) Initial state of coated surfaces before chemical exposure. (**b**) Visual observation after 1 h of contact with corrosive droplets, (**c**) after 24 h of exposure followed by wiping with a wet tissue, and (**d**) after 40 cycles of repetitive application and removal of the corrosive solutions.

To evaluate the chemical durability and corrosion resistance of HMDS-modified PU coatings (G-HMDS-10 wt.%), a comparative chemical resistance test was conducted using stainless-steel coupons coated with the optimized PU formulation containing 10 wt.% HMDS. Uncoated stainless-steel coupons serve as control samples (Figure 11a). The experimental design aimed to simulate harsh chemical exposure conditions and assess the protective efficacy of the coatings against commonly encountered corrosive agents. Three distinct aqueous solutions, such as saturated NaCl solution, 1M NaOH solution (alkaline), and 1M H_2_SO_4_ solution (acidic), were selected as representative corrosive environments. One drop of each solution was carefully placed on both the coated and uncoated regions of the stainless-steel substrates. The samples were left undisturbed for 24 h to allow sufficient interaction between the test solutions and the material surfaces (Figure 11b). Following exposure, the affected areas were gently wiped with wet tissue to simulate routine surface cleaning (Figure 11c). After 24 h of initial exposure, the coated regions exhibited no visible change in surface appearance across all three chemicals. The droplets remained bead-like, suggesting strong hydrophobic behavior and minimal surface interaction. This process was repeated for 25 cycles on each test substrate to examine long-term chemical resistance and surface integrity. Even after 25 repeated cycles of exposure and wiping, the G-HMDS-10 wt.% coated PU films continued to protect the underlying substrate, retaining their structural and aesthetic integrity without any evidence of discoloration, swelling, cracking, or material degradation (Figure 11d). In contrast, the uncoated regions of the stainless-steel coupons showed significant corrosion and surface staining, particularly after prolonged exposure to alkaline (NaOH) and acidic (H_2_SO_4_) environments. The acid-exposed uncoated region darkened and exhibited signs of etching, while the base-exposed area developed noticeable discoloration and surface roughening—clear indicators of chemical attack and material compromise. The salt solution exposed the uncoated area, also showing early signs of surface dulling and minor corrosion after repeated cycling. These results affirm the superior chemical resistance and protective barrier properties imparted by the G-HMDS-10 wt.% PU coatings. The effectiveness can be attributed to the silane’s hydrophobic and low surface energy characteristics, which help resist chemical ingress and reduce surface wettability [42]. The robust performance across neutral, acidic, and basic environments highlights the coating’s versatility for applications in chemically aggressive settings, such as those found in marine, industrial, or biomedical contexts.

### 3.9. Water Contact Angle Test

Surface wettability is a critical parameter in assessing the hydrophobicity and surface energy of coating materials, with direct implications for self-cleaning, stain resistance, and environmental durability. To quantify the wettability of the synthesized PU films, static WCA measurements were performed on CT, glycerol-modified (GLY-10 wt.%), and HMDS-modified (G-HMDS-10 wt.%) PU-coated surfaces. The results are summarized in Figure S5. The control PU film exhibited an average contact angle of 91.28°, indicative of a moderately hydrophobic surface just above the conventional hydrophobicity threshold of 90°. This value reflects the inherent surface chemistry of the PU formed from SOP and MDI, where moderate crosslinking and the presence of unreacted polar groups contribute to limited water repellency. Upon incorporation of 10 wt.% glycerol, the contact angle slightly increased to 93.33°, suggesting improved surface uniformity and reduced polar surface exposure due to enhanced crosslink density. The presence of glycerol leads to the formation of a more compact and less permeable network, which limits the surface expression of -OH groups and contributes to marginally better water repellency. A more pronounced increase in hydrophobicity was observed for the G-HMDS-10 wt.% film, which showed an average contact angle of 95.76°. This enhancement is attributed to the incorporation of HMDS, a low-surface-energy organosilane known for its hydrophobic characteristics. Although HMDS does not chemically bond within the polymer matrix, its preferential migration to the film surface during curing likely results in silane group enrichment at the air–film interface, significantly reducing surface polarity and improving water repellency [58]. These incremental improvements in WCA measurements correlate well with other surface-related tests, such as the ink resistance and stain repellency evaluations, where the HMDS-modified films demonstrated superior performance. The combined evidence underscores the multifunctionality of HMDS as a surface-active additive that not only enhances mechanical integrity and chemical resistance but also substantially improves surface hydrophobicity, thereby expanding the application potential of bio-based PU coatings in harsh and contamination-prone environments.

## 4. Conclusions

In this study, a dual-modification strategy was successfully developed to overcome the inherent trade-off between mechanical robustness and surface durability in bio-based PU coatings. By integrating glycerol as a trifunctional crosslinking agent and HMDS as a silane-based surface modifier, a structurally optimized and functionally enhanced PU system was achieved. The incorporation of glycerol significantly increased the crosslinking density of the SOP-based PU network, leading to a substantial improvement in tensile strength and hardness, with optimum performance observed at 10 wt.% glycerol (47.18 MPa tensile strength and highest hardness). However, excessive glycerol content resulted in network heterogeneity and reduced mechanical performance, highlighting the importance of controlled crosslinking. The subsequent incorporation of HMDS enabled the development of a hydrophobic, silicon-enriched surface without disrupting the underlying urethane network. At an optimal loading of 10 wt.% HMDS, the coatings exhibited a high WCA (95.76°), along with excellent stain resistance, ink repellency, and chemical durability, while retaining considerable mechanical strength (38.19 MPa). Higher HMDS concentrations led to phase separation and reduced interfacial compatibility, confirming that the performance of the system depends on a delicate balance between chemical crosslinking and physical modification. Thermal analysis demonstrated that both glycerol and HMDS contribute to enhanced thermal stability, as evidenced by increased T_g_, char residue, and stable degradation behavior. GC and swelling studies further confirmed the formation of a highly crosslinked and solvent-resistant network, while coating performance tests validated the long-term durability and self-cleaning capability of the HMDS-modified films under repeated exposure conditions. Overall, this work establishes a synergistic design approach in which chemical crosslinking and surface engineering are effectively combined to produce high-performance, bio-based PU coatings. The developed system not only addresses key limitations of conventional bio-based PUs but also offers a scalable and fluorine-free pathway toward sustainable coatings with advanced mechanical integrity and surface functionality. These findings provide a practical framework for designing next-generation eco-friendly coatings for industrial and protective applications.

## Figures and Tables

**Table 1:** Comparative analysis of previously reported bio-based PU coating materials.

Sr. No.	Bio-Based Material	Cross-Linker or Additives	Mechanical Strength	Thermal Properties	Coating Test	Ref.
1	Castor oil	Monocarbinol-terminated PDMS−OH	Nanoindentation hardness—657 MPa, Pencil hardness—6H	-	WCA—103.2°, Water sliding angle—33.8°, Oil-based marker residual area—16.4%, Water-based marker residual area—5.4%	[38]
2	Castor oil	Monocarbinol-terminated PDMS−OH	Pencil hardness—3H	-	WCA—105.8°, Sliding angle—20.6°	[39]
3	Pine alkali lignin	Monocarbinol-terminated PDMS−OH	Hardness—260.1 MPa, Elastic modulus—4.7 GPa	-	Water & Hexadecane-: contact angle- 105° & 30°, Slide Angle—49° & 11°	[40]
4	Cottonseed oil Sebacic acid Succinic acid Maleic acid Azelaic acid	Tartaric acid	Pencil hardness—5H, Gloss value 60°–119°	T_g_—60–81 °C	WCA—133°	[41]
5	SOP	DPSD	Hardness (D)—75	T_MAX_—470 °C, T_g_—50.98 °C	WCA—93.93°	[42]
6	Castor oil-	2,2-di (hydroxymethyl) propionic acid	Elongation—41.40%, Tensile strength—22.21 MPa	T_MAX_—482.88 °C	WCA—68.19°	[43]
7	Acetylated starch (ac Starch)	Monocarbinol-terminated PDMS−OH	Hardness—3H		WCA—107.8°, Water slide angle—34.5°	[44]
8	Itaconic acid	2-hydroxyethyl methacrylate	Pencil hardness—6H, Impact resistance intrusion—112, Impact resistance extrusion—74	T_g_—62.03 °C, Residues at 700 °C—4.78%	GC—99.39%, Water absorption—0.80%	[45]
9	castor oil	Itaconic acid acrylate, ricinoleic acid acrylate and oleic acid acrylate	Pencil hardness—6H, Tensile strength—22.71 MPa, Elongation—11.24%, Young’s modulus—202.05 MPa	T_g_—75.62 °C, T_MAX_—425.5 °C	GC—98.57%	[46]
10	Castor oil	Pentaerythritol tri-acrylate	Pencil hardness—6H, Tensile strength—12.32 MPa, Elongation—11.88%	T_g_—72.1 °C, T_MAX_—474.3 °C	GC—99.27%	[47]
11	Tung oil	N-methyl diethanol amine, polyethylene glycol	Tensile strength—15.8 MPa, Elongation—80.2%, Young’s modulus—382.1 MPa, Pencil hardness—4H	T_g_—60.7 °C, T_MAX_—414.2 °C	WCA—100.2°, Water absorption—4.9%	[48]
12	Enzymatic hydrolysis of lignin	-	Pencil hardness—3H, Tensile strength—81.6 MPa, Elongation—8.1%, Young’s modulus—1399 MP	T_g_—112 °C, T_MAX_—417.1 °C	WCA—87.6°, Anticorrosive E_corr_—(118 mV)	[49]
13	5-Hydroxymethylfurfural	-	Pendulum hardness (S)—189, Reverse impact(in·lb)—16, Nanoindentation hardness—473.5 MPa, elastic modulus—6.03 GPa	T_g_—177 °C, char yield—19%	-	[50]
14	Methacrylate monomer	Maleic anhydride	Pencil hardness—6H, Tensile strength—22.7 MPa, Strain—7.2%, Young’s modulus—3293.5 MPa	T_g_—109.2 °C, T_MAX_—438 °C	-	[51]
15	SOP	Glycerol, HMDS	Tensile strength—38.19 MPa, Hardness—48.5	T_g_—74.19 °C, T_MAX_—470 °C, residual mass—21.98%	WCA—95.76°	[This work]

**Table 2:** Thermal characteristics of PU samples.

Sr. No.	Sample Name	T_5%_ (°C)	T_MAX_ (°C)	T_g_ (°C)	Residual Mass (%)
1	CT	309	387	28.06	18.52
2	GLY-5 wt.%	300	474	71.45	21.0
3	GLY-10 wt.%	293	470	76.69	20.9
4	GLY-15 wt.%	287	171	82.62	24.70
5	GLY-20 wt.%	285	465	85.23	26.66
6	G-HMDS-10 wt.%	290	470	74.19	21.98

## Data Availability

The original contributions of this study are included in the article/Supplementary Material. Further inquiries can be directed to the corresponding authors.

## Supplementary Materials

The following supporting information can be downloaded at: https://www.mdpi.com/article/10.3390/polym18121490/s1, Table S1: Characteristic data of SO, ESO and SOP; Table S2: Formulation table of the synthesis of glycerol-based PU samples (GLY-Xwt.%); Table S3: Formulation table of the synthesis of HMDS-based PU samples (G-HMDS-Xwt.%); Table S4: Viscosity and mechanical test data of synthesized PU materials; Figure S1: (a) FTIR (b) GPC of SO, ESO and SOP (c) ^1^H NMR of SOP; Figure S2: Tensile strength with error bars of (a) glycerol-based PU samples (GLY-Xwt.%), (b) HMDS-based PU samples (G-HMDS-Xwt.%); Figure S3: (a) TGA & DTGA of G-HMDS-10wt.% (b) DSC spectra of HMDS-based PU samples (G-HMDS-Xwt.%); Figure S4: Visual comparison of dispersion and drying behavior of films with varying weight percentages of glycerol and HMDS in water and toluene solvents (a, c, e, g) show images of samples immersed in different solvent systems (water or toluene) for 24 h. (b, d, f, h) show the corresponding films after solvent removal and drying at 90 °C for 24 h; Figure S5: water contact angle measurements of films indicating surface wettability characteristics.
