# Supplementary material for: Synergistic Role of Crosslinker and Silane-Based Additive in Designing Structurally Robust Bio-Based Polyurethane Coatings
Source: Polymers (Basel). 2026 Jun 13;18(12):1490. doi: 10.3390/polym18121490 (PMC13306967; doi:10.3390/polym18121490)
Supplement: Supplementary file 1 [file polymers-18-01490-s001.zip › polymers-4226102-supplementary.pdf]

Synergistic Role of Crosslinker and Silane-based Additive in Designing Structurally Robust Bio-based Polyurethane Coatings

Mayankkumar L. Chaudhary[1]*, Kinal Chaudhari[1, 2], Rutu Patel[1] and Ram K. Gupta[1,3]*

[1]National Institute for Materials Advancement, Pittsburg State University, 1204 Research Road, Pittsburg, KS 66762, USA
[2]Department of Physics, Pittsburg State University, 1701 S Broadway St, KS, 66762
[3]Department of Chemistry, Pittsburg State University, 1701 S Broadway St, KS, 66762
*Corresponding author: mayank.chaudhary1807@gmail.com; ramguptamsu@gmail.com

Equation S1. EOC (%) = $\frac{V \times N \times 1.6}{Wt.\ sample}$

Equation S2. $GC\ (\%) = \frac{m_2}{m_0} \times 100$

Where m_2 is the weight after drying.

Equation S3. $DS\ (\%) = \frac{m_1 - m_0}{m_0} \times 100$

Where $m_0$the initial is weight and m_1 is the weight after swelling.

Table S1. Characteristic data of SO, ESO and SOP

Test	Units	SO	ESO	SOP
**Iodine value**	**gI_2/100g**	132.27	0.27	-
**Epoxy-oxirane oxygen value**	**%**	-	7.41	0.22
**Hydroxyl value**	**mg KOH/g**	-	-	181
**Acid value**	**mg KOH/g**	0.17	1.29	1.10
**Viscosity**	**Pa.s**	0.022	0.17	2.38

Table S2. Formulation table of the synthesis of glycerol-based PU samples (GLY-Xwt.%)

	CT	GLY-5wt.%	GLY-10wt.%	GLY-15wt.%	GLY-20wt.%
**SOP**	5	5	5	5	5
**Glycerol**	0	0.25	0.50	0.75	1
**MDI**	2.28	3.38	4.48	5.58	6.69

Table S3. Formulation table of the synthesis of HMDS-based PU samples (G-HMDS-Xwt.%)

	G-HMDS-10wt.%	G-HMDS-20wt.%	G-HMDS-30wt.%	G-HMDS-40wt.%	G-HMDS-50wt.%
**SOP**	5	5	5	5	5
**Glycerol**	0.50	0.50	0.50	0.50	0.50
**HMDS**	0.99	1.99	2.99	3.99	4.99
**MDI**	4.48	4.48	4.48	4.48	4.48

Table S4. Viscosity and mechanical test data of synthesized PU materials

Sample Name	Viscosity (Pa.s)	Tensile strength (MPa)	Hardness (D)
**CT**	2.6	6.7	32.16
**GLY-5wt.%**	-	27.49	50.5
**GLY-10wt.%**	2.2	47.18	67.5
**GLY-15wt.%**	-	33.34	57.73
**GLY-20wt.%**	-	19.66	48.33
**G-HMDS-10wt.%**	1.7	38.19	48.5
**G-HMDS-20wt.%**	-	32.52	35.5
**G-HMDS-30wt.%**	-	32.03	31
**G-HMDS-40wt.%**	-	25.4	26.5
**G-HMDS-50wt.%**	-	20.98	21.5

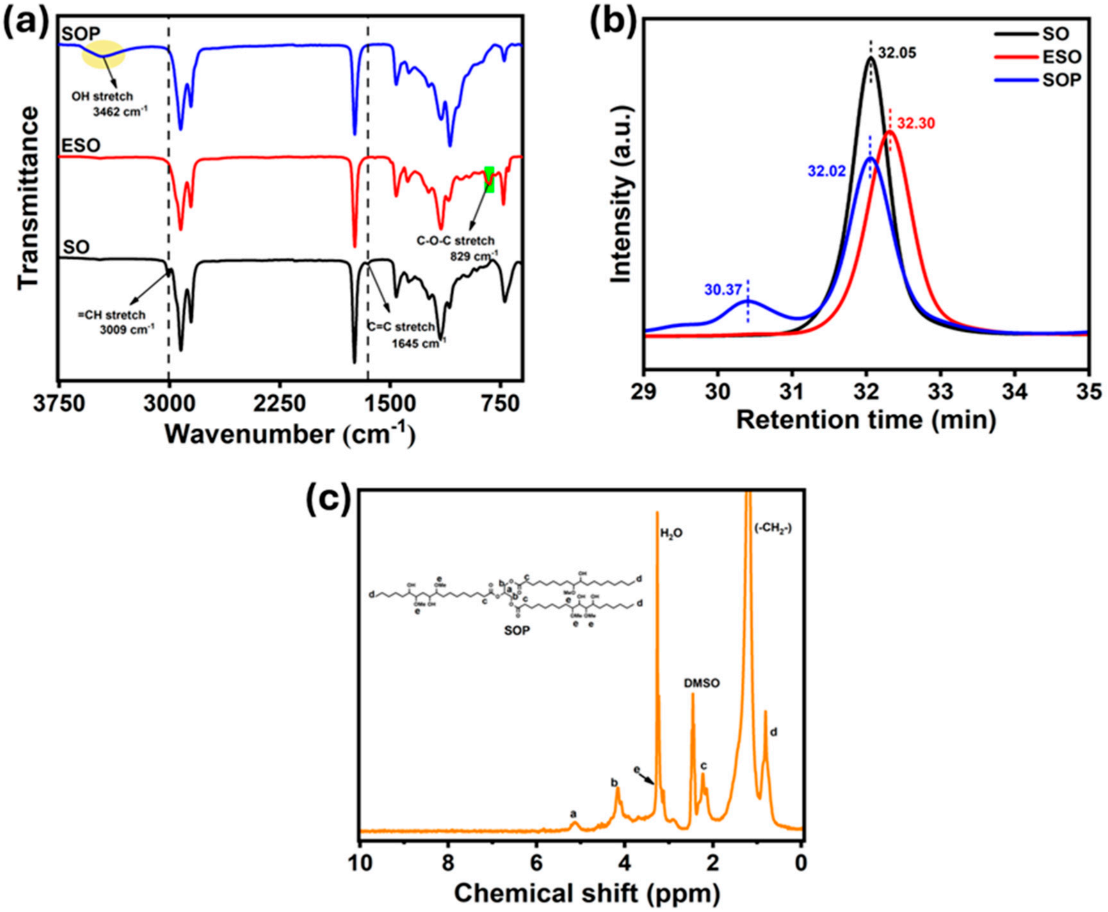

Figure S1. (a) FTIR (b) GPC of SO, ESO and SOP (c) ^{1}H NMR of SOP

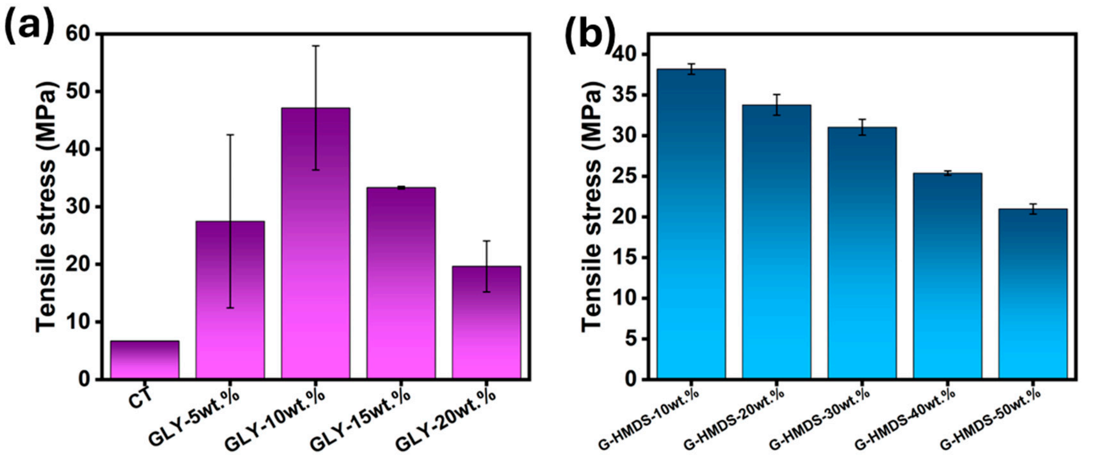

Figure S2. Tensile strength with error bars of (a) glycerol-based PU samples (GLY-Xwt.%), (b) HMDS-based PU samples (G-HMDS-Xwt.%)

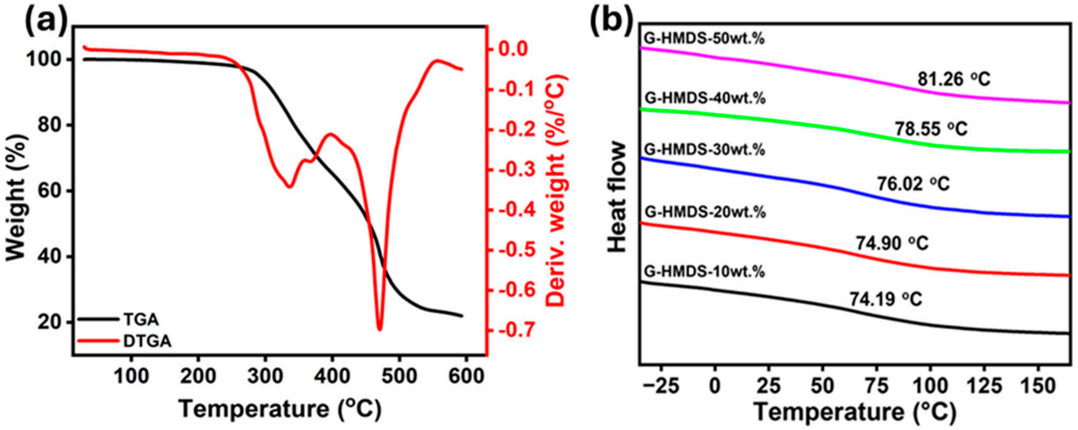

Figure S3. (a) TGA & DTGA of G-HMDS-10wt.% (b) DSC spectra of HMDS-based PU samples (G-HMDS-Xwt.%)

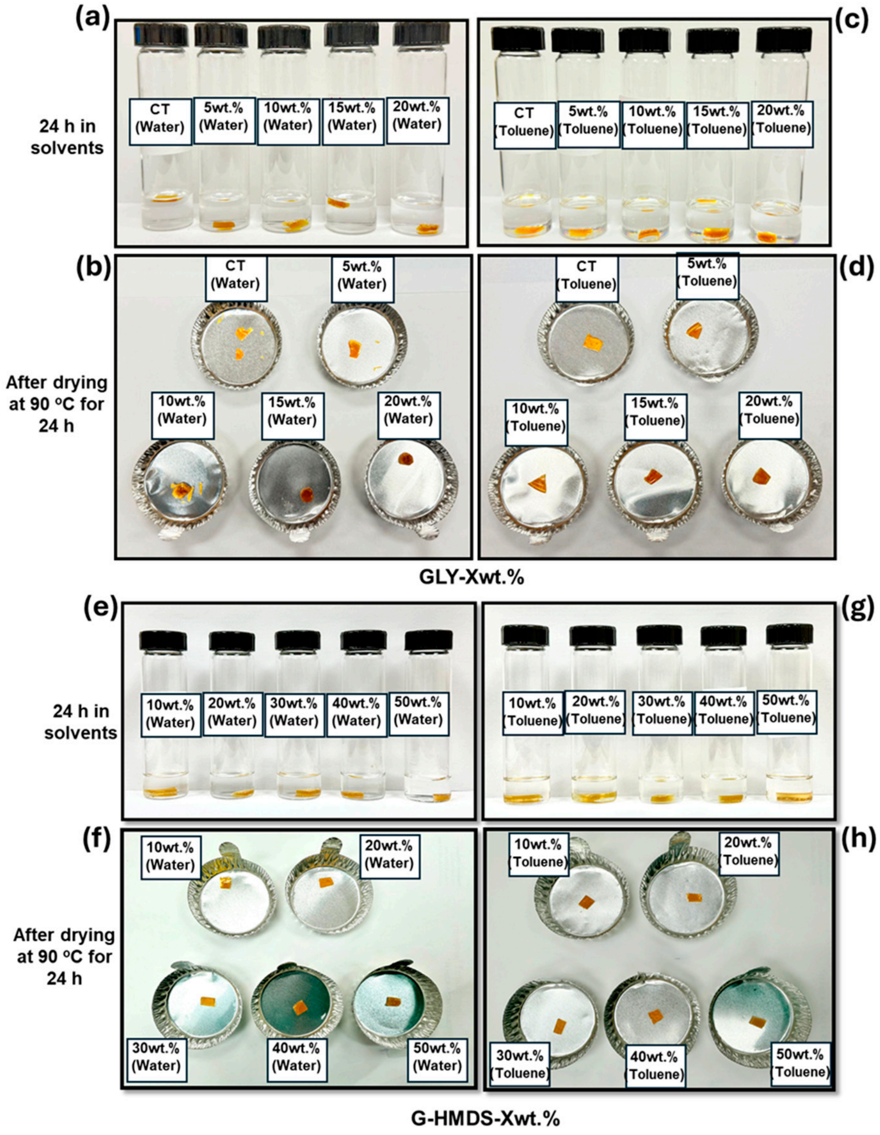

Figure S4. Visual comparison of dispersion and drying behavior of films with varying weight percentages of glycerol and HMDS in water and toluene solvents (a, c, e, g) show images of samples immersed in different solvent systems (water or toluene) for 24 hours.
(b, d, f, h) show the corresponding films after solvent removal and drying at 90 °C for 24 hours.

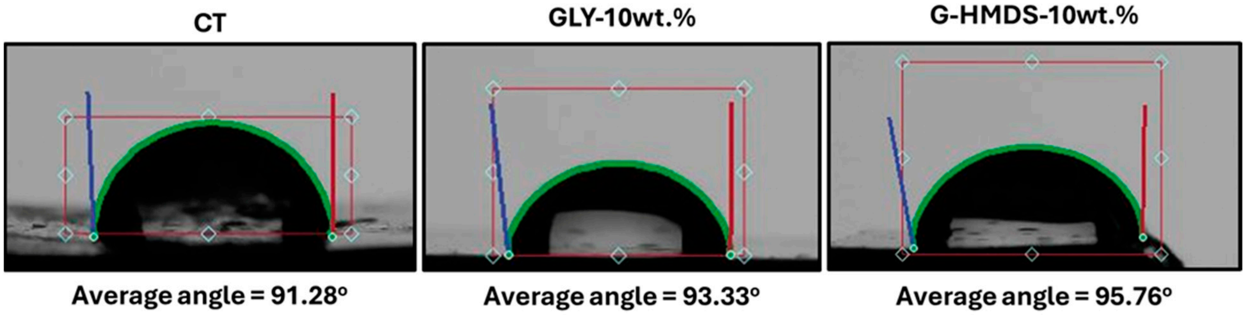

Figure S5. water contact angle measurements of films indicating surface wettability characteristics
